# A research agenda for the study of social norm change

**DOI:** 10.1098/rsta.2020.0411

**Published:** 2022-07-11

**Authors:** Giulia Andrighetto, Eva Vriens

**Affiliations:** ^1^ Institute of Cognitive Sciences and Technologies, National research Council of Italy, via Palestro 32, 00185 Rome, Italy; ^2^ School of Education, Culture and Communication, Division of Mathematics and Physics, Malardalens University, 883, 721 23 Västerås, Sweden; ^3^ Institute for Future Studies, Holländargatan 13, 111 36 Stockholm, Sweden

**Keywords:** social norms, norm change, tipping points, experiments, agent-based modelling, computational social science

## Abstract

Social norms have been investigated across many disciplines for many years, but until recently, studies mainly provided indirect, implicit and correlational support for the role of social norms in driving behaviour. To understand how social norms, and in particular social norm change, can generate a large-scale behavioural change to deal with some of the most pressing challenges of our current societies, such as climate change and vaccine hesitancy, we discuss and review several recent advances in social norm research that enable a more precise underpinning of the role of social norms: how to identify their existence, how to establish their causal effect on behaviour and when norm change may pass tipping points. We advocate future research on social norms to study norm change through a mechanism-based approach that integrates experimental and computational methods in theory-driven, empirically calibrated agent-based models. As such, social norm research may move beyond unequivocal praising of social norms as the missing link between self-interested behaviour and observed cooperation or as the explanation for (the lack of) social tipping. It provides the toolkit to understand explicitly where, when and how social norms can be a solution to solve large-scale problems, but also to recognize their limits.

This article is part of the theme issue 'Emergent phenomena in complex physical and socio-technical systems: from cells to societies'.

## 1. Introduction

Social norms have gained attention as solutions to some of the most pressing social and environmental challenges of our contemporary societies, such as climate change and vaccine hesitancy [1,2]. As informal, non-codified, social rules that regulate behaviour, they have a long history governing stable practices in society such as tipping, the food we eat and how we talk to each other. Yet (emerging) social norms may also promote behavioural change: the spread and evolution of social norms contributed in a crucial way to, for example, the reduction of smoking in public places [3], the increase in eco-friendly behaviour [4–6], the change in food consumption and eating behaviour [7] and the adherence to social distancing in response to the COVID-19 pandemic [8,9].

These challenges have in common collective action problems where groups benefit from collective behaviour, but individuals' incentives drive them to free ride on others' efforts. While interventions to address these collective action problems could be taken through formal institutions like laws and treaties, the large-scale and rapid development of these challenges often make formal institutions insufficient, unenforceable or too costly [2,10]. For example, to reduce the carbon footprint, governments may invest in green technologies like sustainable energy and hybrid vehicles, but this will not be effective unless citizens also choose to adopt them [11]. This thus renders informal institutions, and in particular social norms, essential [12–15].

Social norms have several properties that make them well suited to solve collective action problems [14,16,17]. First, social norms greatly increase the predictability of people's behaviour [18] and thereby enable social coordination [2]. So indispensable to social life, they are defined as the ‘cement’ [19], the ‘grammar’ [13] or the ‘glue’ [20] of society. Second, when enforced through mechanisms such as gossip, ostracism and peer punishment, social norms can motivate individuals to act against their self-interest [21,22]. Third, they provide simple instructions on how to behave and indicate what behaviours are effective in achieving the common goal, without needing to completely understand the often complex collective action problem [23]. Finally, norms help to coordinate and stabilize behaviour also under changing macro conditions [24]. Societies with strong social norms are, for instance, better able to informally coordinate behaviour under collective threats, like climate change and pandemics [8,16].

While social norms have long been of interest to scientists of a wide variety of disciplines, empirical evidence of the causal relationship between a norm and behaviour is rare. In fact, most social norm research takes the effect of social norms for granted and uses norms as a post hoc interpretation of observed phenomena [25]. Being outcome-oriented, it seeks to answer the question ‘Does intervention X change behaviour Y’? While this allows for a rapid transfer of research to policy and implementation, this approach strongly hinders generalizing the effect that an intervention has in changing a behaviour, because the way in which the intervention works is largely left unexamined. Besides intuitions, there is little theoretical basis upon which to rest the predictions for the applicability of a specific strategy on a new set of phenomena. To fully understand the potential of these informal rules for guiding and changing behaviour, social norms need to be explicitly measured and the mechanisms by which norms are hypothesized to cause behaviour need to be assessed rigorously; especially if we want to identify ways in which norms can be changed (spontaneously or intentionally).

To make progress in identifying the conditions under which social norms emerge, develop and shift, and generate large-scale behavioural change, we believe that an interdisciplinary and multi-method approach that integrates experimental and computational methods is needed. Behavioural experiments help to test the causal effect of social norms on individual behaviour and identify factors leading social norms to change [26]. Large-scale, agent-based simulations allow investigating the conditions for norm change to spread from small groups to large ones [27].

A rigorous, mechanism-based approach is possible due to several recent advances in the study of social norms. While a systematic review of these advances is beyond the scope of this paper (for an extensive review see [28]), we highlight insights from recent developments in social norms literature that enable a more integrated research agenda for the study of social norms in the future. In the next sections, we discuss the main conceptual and methodological advances starting with (i) a clear and operational definition of social norms that distinguishes them from, e.g. conventions, moral norms and behavioural regularities; (ii) a discussion of the new measurement techniques to assess the existence of norms and their causal effect on behaviour; and (iii) an overview of tipping point models to understand norm change. Subsequently, we propose a bridge between these largely independent research lines in a future research agenda for social norms and norm change that encompasses (i) the use of empirical data to calibrate and validate dynamic computational models; (ii) more attention for contextual and individual heterogeneity; and (iii) a critical reflection on the limits of social norms as a means to solve large-scale problems.

## 2. Recent advances in the study of social norms

### (a) What are social norms?

Following Bicchieri [13], we define social norms as informal and shared behavioural rules that prescribe what one ought or ought not to do that people comply with because of social expectations and potential social sanctions. On this account, compliance with social norms is *conditional* on ‘empirical expectations’, i.e. the belief that a sufficiently large number of people in their community conform to the rule, and ‘normative expectations’, i.e. the belief that a sufficiently large number of people in their community think that they ought to conform to the rule and may be willing to sanction transgressions. In general, empirical expectations about the prevalence of a behaviour are crucial, but not necessarily sufficient for norm following. In situations where individual and social interests align, but a coordination problem needs to be solved (such as driving on the right side of the road), empirical expectations may suffice. However, when social norms prescribe behaviour that goes against narrow, self-interested motives, like in collective action problems, the associated costs may make self-interested individuals uninterested in norm compliance regardless of their empirical expectations—in fact, it provides an incentive to free ride. In these situations, empirical expectations need to be complemented by normative expectations, for these often come with the expectation of social sanctions [29]. Similarly, normative expectations should be met with empirical expectations, since otherwise people may see that norms can be violated without sanctions.

This definition that decomposes aggregate social norms as the result of individuals' expectations has several advantages. It allows us to distinguish social norms from other related concepts. Differently to habits (e.g. brushing teeth in the morning), recurrent behavioural patterns (e.g. people putting up umbrellas at the same time when it rains) and moral norms (e.g. not harming innocent people) that are primarily unconditional or driven by non-social motives, compliance with social norms is conditional on social expectations. This definition also allows us to separate social norms from conventions or descriptive norms whose compliance instead is only motivated by expectations about others' actions (i.e. empirical expectations) and lacks the normative ‘ought’ component [13,18].

Moreover, by translating macro-level social norms into micro-level expectations, the presence of social norms and their causal effect on behaviour can be empirically assessed rather than post hoc assumed. That is, behaviour and corresponding social expectations signal the presence of social norms; their causal effect can be assessed by testing whether by changing expectations, a change in behaviour follows. Once we have determined that a behaviour is sustained by a social norm, it becomes possible to identify interventions to generate norm change. If people are motivated not only by personal normative beliefs (i.e. one's own belief about what ought to be done), but also by a preference to do what others are doing (i.e. empirical expectations) and what others think should be done (i.e. normative expectations), norm-based interventions may generate a change in behaviour by modifying people's (empirical and/or normative) expectations—which is arguably much easier than changing their personal normative beliefs [30,31].

Finally, this micro-level definition of social norms also enables a better assessment of the strength of social norms at the macro-level. Knowing the overall strength of a social norm improves predictions of its impact on behaviour. Specifically, it has been suggested that aggregate social norm strength can be inferred by comparing the social expectations of group members with respect to their internal consistency, accuracy and specificity [24]. That is, the higher the agreement between expectations of group members (consistency), the better group members are at predicting the behaviour and personal normative beliefs of others (accuracy), and the closer the individuals' social expectations are to the group's average (specificity), the stronger the social norm. The higher the norm strength among people of the same group, the more established the social norm, and the stronger its effect on behaviour.

### (b) Methods for testing the causal effect of social norms

Researchers studying social norms often show that their results are correlationally and implicitly consistent with social norm-based explanations. For instance, social norms are typically posited to explain behaviour when that behaviour is incompatible with the self-interested solution to a game [14,22]. For instance, peoples' willingness to punish others when punishment is costly and brings no future benefits (e.g. in one-shot interactions) is explained through the presence of social norms [32,33]. Yet this is an indirect inference of social norms and does not distinguish whether the decision is motivated by social norms or by other factors, such as individual preferences that result in similar outcomes.

Recent research has made progress in that regard by putting social norms to a more rigorous test that measures their presence directly and assesses whether they causally influence the studied phenomenon [6,24,29,34–36]. Different belief-elicitation protocols have been developed to estimate whether individuals hold sufficiently high and consistent empirical and normative expectations, and hence to determine whether a majority believes that a norm applies in a given situation. Bicchieri & Chavez [37] developed a survey method where subjects are presented with a variant of the Ultimatum Game in which they are asked to indicate what actions they consider as fair (personal normative beliefs), how they expect others to behave in this situation (empirical expectations) and what actions they think would be considered as fair by most participants (normative expectations). To incentivize precision in assessing social expectations, subjects are paid on the basis of their accuracy in guessing the expectations of others. Krupka & Weber [36], instead, elicit social norms using a coordination game. Subjects observe a hypothetical Dictator Game (0–10) and are asked to guess modal appropriateness ratings of different actions (likewise incentivized). This method is used to verify the presence of shared normative expectations, but does not inquire about personal normative beliefs. For an extended version of this approach, see [38].

Measuring the existence of social norms is important, but not sufficient to claim that these are causally driving a particular behaviour. To this end, instruments were developed that test for a causal role of social norms on the observed behaviour [36,38]. In vignette surveys, respondents are presented with hypothetical situations, for which the information about the behaviour and normative beliefs of others are manipulated, such that respondents will update their empirical and normative expectations. If the manipulation results in a change in behaviour, this is considered evidence of the causal effect of the social norm on the behaviour under study.

This mechanism-oriented work on the measures and manipulations of social norms is one of the more interesting developments within recent studies on social norms. Yet, one shortcoming is that it mainly relies on short-term laboratory experiments. These are suitable for measuring the existence of norms and their causal effect on behaviour, but not ideal for studying the evolution and change of norms over time. This is a serious limitation, since understanding which conditions allow norms to emerge, spread and change, is crucial for identifying interventions which could lead to large-scale behavioural change. Recently, a 30-day online experiment was conducted to test how norms develop over time [24]. Subjects were asked daily to make decisions and indicate their personal normative beliefs as well as empirical and normative expectations to identify social norms, establish their causal effect on behaviour and measure their change over time. Compared with standard short-term designs, a longer term experiment allowed studying the emergence, spread and change of social norms, on top of their effect on behaviour.

All in all, the mechanism-based behavioural experiments have proven useful in understanding the causal relation between norms and behaviour in general, but they remain limited in scalability and generalizability. Even with longer experiments, the time available for norms to emerge, develop and change remains limited. Moreover, experiments often test social norms or norm change for small groups and under specific experimental conditions. It is not evident that the dynamics would be similar in large societies.

### (c) Tipping points for social norm change

Unlike formal institutions and top-down regulations, social norms often strengthen or weaken over time, because what is considered appropriate is subject to change [16]. Think, for instance, of the widespread norm shift from normalizing to discouraging smoking in public places, of the change in dominancy from norms against same-sex marriage to norms favouring same-sex marriage in many countries and of the sudden inappropriateness of the age-old social norm of handshaking following the outbreak of the COVID-19 pandemic. Sometimes norm change is triggered by changes in legislation or (sudden) environmental change, but often norm change occurs spontaneously [39,40]. Even when top-down interventions trigger norm change, these interventions—acting as catalysts—are themselves the result of changing collective attitudes and awareness and growing social pressure [41].

What makes social norm change difficult to predict, however, is that often it is not a gradual or even a conscious process [26]. There may be shared reasons to change, but that does not necessarily translate into a collective change in social expectations and in coordinated action. In collective action problems, being a first (or early) mover is often costly despite shared reasons to change. A popular framework to understand when norm change *does* occur is through the notion of tipping points [2,42,43]. It posits that change, even when desirable, often fails to be achieved unless a tipping point is reached. The tipping point reflects a threshold, lower than a 51% majority, where the proportion of people deviating from an existing norm (or complying with a new norm) becomes large enough that the majority's expectations about what is socially acceptable suddenly change [39]. That is, once the tipping point is reached, even those who are risk averse, conformist or wary of change have an incentive to follow [44,45].

To reach tipping points a small minority of committed individuals should be willing to transgress existing norms [44]. These committed individuals have a low threshold for change and do not let their behaviour be affected by opposing social expectations or social sanctions [46]. They generally have strong personal normative beliefs in favour of the new, desired social norm (i.e. high norm sensitivity), are autonomous in their decision making and are less sensitive to the (perceived) risk of deviating from an established norm. By (repeatedly) demonstrating behaviour in disagreement with the existing social norm, they may slowly change empirical expectations of others, who start observing more norm violations and—depending on their personal norm and risk sensitivity—may or may not be willing to follow suit. Then, once the tipping point is reached, large-scale change in both behaviour and normative expectations follows [42]. In other words, while early movers act out of (a change in) their personal normative beliefs, after passing the tipping point the large majority follows not because of a change in their personal normative beliefs, but because of a change in social expectations.

The crucial question is, thus, how many people have to adopt a new norm before tipping occurs and social incentives reverse. Unfortunately, social tipping is rarely anticipated and usually only understood in hindsight [44]. Such post hoc observations are of limited explanatory value. If it were possible to locate a tipping point, not only would it be possible to predict when it will be reached, but interventions may be designed not with the (ambitious) aim of changing everyone’s personal normative beliefs, but merely to flip enough first movers to reach the tipping threshold where social expectations reverse and large-scale behavioural change follows [2].

Using computational models, various scholars have provided estimates of how many committed individuals are needed to pass a tipping point, mostly with respect to social conventions (i.e. for which empirical expectations suffice). The Naming Game [47], for instance, models the emergence and abandonment of conventions by randomly matching agents who have to agree on a name for an unknown object. Through simulations, stabilization (i.e. name consensus) is predicted based on, for example, the percentage of committed individuals, the range of potential names and the interaction structure. Theoretical tipping point predictions range from minorities as large as 40% of the population [48] to as small as 10% [46] or even 3% [49].

Behavioural experiments that exogenously manipulate the percentage of committed individuals have tested the occurrence of social tipping for real (yet controlled) interactions. Using a variation of the Naming Game, it has been shown empirically that collective agreement emerges quickly and spontaneously [50]. In a follow-up experiment, tipping points were sought by introducing, after agreement was reached, committed minorities of varying sizes that attempted to overturn the established agreement with a novel alternative. Committed minorities smaller than 25% were never successful, but for minority sizes of 25% and larger the established agreement was always overturned [51].

In a different experiment, the passing of a computationally derived tipping point at 35% was tested in a social dilemma setting where participants have to choose between Blue or Green and earn points when they coordinate on the same colour [44]. At the start, the value of Blue exceeded that of Green for everyone, creating a clear preference and a social norm of choosing Blue. Over time, for some participants, the value of Green was changed to exceed that of Blue, which gave them a personal incentive to deviate from the norm of choosing Blue. The simulations correctly predicted the presence or absence of tipping for 23 of 24 experimental societies.

These studies—both computational and experimental—provide support for the idea that social change often occurs through tipping point dynamics. Instead of a gradual, linear change, collective behaviour follows an S-shaped curve where the majority of people change their behaviour well before 50% of the population adopt a new behavioural alternative. However, tipping point models to date are usually applied to social conventions, without explicitly modelling and measuring the social mechanisms driving the change. Yet people can change their behaviour for a variety of reasons. Other than because of social expectations (empirical and/or normative), they may fear social sanctions or may change their behaviour because of changes in their personal preferences and beliefs. While one could argue that whether or not norm change drives the behavioural change is irrelevant as long as the outcome is achieved, this does not hold when the goal is to *reach* the tipping point.

When designing interventions to move a large enough minority to the tipping point, the success depends on identifying and targeting the right mechanisms driving behaviour. As long as tipping point models follow an abstract descriptive and predictive approach with little attention paid to the mechanisms through which the outcomes are brought about, it is difficult to explain why we observe what we observe [52]. This inhibits, among others, the generalization of the results to other contexts and the derivation of broader implications for the predicted behavioural patterns. Thus, when the aim is to reach the tipping point, we need not only to locate it, but also to diagnose what sustains the dominant behaviour. For instance, if preferences rather than social norms drive behaviour, social norm interventions are not effective [53]. But if it is indeed social expectations underlying the decision making, those expectations may be changed through the toolkit of norm-based interventions.

## 3. A research agenda for the study of social norm change

In recent years, research on social norms and how they can generate social change has advanced greatly through the introduction of (i) a micro-level definition of social norms, (ii) methods that allow to measure their causal effect on behaviour and (iii) computational models to derive tipping points for (social norm) change. However, these fields so far developed largely independent of each other. Below we present three directions for future research on social norm change that rely on the integration of these research lines. This integration would further advance understanding of how social norms work and how they can be used to generate desirable behavioural change.

### (a) Empirically based computational models of social norms

Social norms are emergent features of agent interaction, so if the goal is to predict, explain and generate norm change, any study should involve an explicit model and test of the evolution of social norms and their strength. The analysis of such patterns requires in-depth integration of different methods, observation scales and disciplines. In particular, understanding the emergence and change of social norms would greatly benefit from research that combines experimental and computational methods, for instance in theory-driven, empirically calibrated agent-based models (for a general introduction to the use of (empirically driven) computational models in the social sciences, see [27,52,54–57]).

Computational tipping point models currently provide little justification for why certain individuals do or do not adopt new norms or conventions or abandon old ones. The different tipping points derived, follow from variations in the chosen parameter space. However, the results are of little practical relevance to understand and explain the presence or absence of norm change from one instance to another as long as this parameter space is not motivated by empirical data. Behavioural experiments, on the other hand, suffer important practical limitations in terms of scalability. Whether it concerns group size (or the complexity of network structures), time or the number of counterfactuals, behavioural experiments are limited with regards to the type of settings for which norm change can feasibly be tested.

Empirically based agent-based models, by combining the benefits of both approaches, offer several advantages that enable research on social norms and norm change to move forward. On the one hand, behavioural experiments provide sound and clear data on agent behaviour, on which to build informative micro–macro models. Being empirically defined, the strategies used by agents are more plausible than what is standard for social science research based on formalized models. On the other hand, agent-based models are able to extend experimental data and scale up (both in number and time) the processes of interactions of a few agents to interactions among many agents over time. Agent-based modelling is well suited to capture multi-level dynamic processes of the individual, group and structure [12], not only by modelling how these processes jointly bring about norm change, but also by allowing these new norms, once emerged, to feedback on individual-level social expectations and decision making—a process often called ‘downward causation’ [58] or ‘immergence’ [59].

In the last years, an increasing number of scholars are starting to develop more cognitively plausible and empirically calibrated agent-based models to examine the emergence and evolution of large-scale, complex social patterns, such as social norms, collective opinions and institutions [56,60–65]. These models are built with the goal of reproducing data from behavioural experiments. By comparing models with and without social norms in the extent to which they reproduce the cooperation dynamics observed in experiments, the empirically calibrated agent-based models give insight into the plausible mechanisms driving behaviour. Of particular interest are empirically calibrated agent-based models that incorporate social norm parameters dynamically, i.e. with agents updating their social expectations based on (observed) behaviour. In Realpe-Gómez *et al*. [65], for instance, decision making is based on the agents' previous actions, their beliefs about others' actions (empirical expectations) and some psychological processes governing their thinking (cognitive consistency). If the results of experiments are consistent with simulation outcomes wherein social norms are explicitly modelled this provides support for the plausibility of the theory embedded in the simulation, and evidence of the effect of social norms in promoting cooperative behaviour.

These evidence-based rich computational models are viable tools for the study of social norms as they can be used as a virtual laboratory to conduct social simulations to scale up the analysis and assess the aggregate consequences for social and behavioural change in different contexts, and to develop future scenarios identifying factors that should be targeted to promote significant behavioural change. In future research, the challenge will be to integrate more and more of these models with empirical work.

### (b) Tightness–looseness and heterogeneity in norm change

Tipping point models suggest that when social norm change occurs in one setting, but not in another, the tipping point was reached only in the former. As an explanation, this is scarcely informative. To understand the potential for and occurrence of social norm change, heterogeneity in both individual and contextual factors needs to be accounted for. Individual heterogeneity, to some extent, is inherent to the tipping point model insofar as different individuals have different thresholds for change [42,43] related to, for instance, their norm and risk sensitivity [36].

There are, however, also considerable cross-cultural and cross-country differences in levels of cooperation, the strength of social norms and the willingness to enforce these norms [16,32,66,67]. According to the tightness–looseness theory, for instance, tight social norms and stricter punishment develop as a function of threat. Cross-country variation in the strength of social norms and punishment is explained by the amount and severity of ecological and human-made societal threats that societies experienced throughout history. Threats, be they related to resource scarcity, natural disasters, territorial attacks or the spread of infectious diseases, require order and social coordination, thereby increasing the need for strong norms and norm enforcement [16]. The hypothesis that social norm and punishment strength is to the threats that nations have (or have not) historically encountered is well supported by evidence from cross-sectional surveys [68], ethnographies [69], computational models [70] and behavioural experiments [24].

The threat history of societies (and thus their respective tightness) is likely to affect the potential for norm change. Yet both theoretically and empirically it remains to some extent an open question how norm change and social tipping may differ across societies. Or, in other words, which contextual conditions favour norm change. In general, threats have the effect of tightening social norms in all societies. In response to sudden threats, populations may rapidly establish and strengthen social norms [24,70]—introducing threats as potential catalysts for moving populations beyond the tipping point. Yet tight and loose societies may not change in the same way or at the same speed. Tight societies may, for instance, quickly strengthen norms and coordinate behaviour if the situation requires it, because they already have stronger social norms in place. Countries with low levels of cultural tightness faced, for instance, substantially higher numbers of COVID-19 cases and deaths compared with countries with high levels of cultural tightness [8]. At the same time, countries that do not already have a history of tightness may also benefit from their loose cultures, when these provide the fertile ground for innovation and creativity that enable new norms to develop and to create turnover [20]. By integrating social norm strength and tightness–looseness into tipping point models of social norm change, these differences may be unravelled further. Future computational models should incorporate such cross-cultural variation that can be derived from survey, field and experimental evidence to understand how norm change and social tipping may be reached across contexts (e.g. [71]).

### (c) The limits of social norms

When clearly defined and put to rigorous empirical test, social norms represent a reliable and accessible mechanism to create large-scale changes in expectations and behaviour [2,14,72]. It is, however, too simplistic to present social norms uniquely as a solution [25]. While they may promote massive transformation of behaviour, under some conditions, they can also be an obstacle to change.

Often the behaviours that contribute to the socially undesirable practice to be changed are themselves the norm. Think for example of activities that contribute to global warming, such as eating meat, using air conditioning, not adopting renewable energy solutions, flying or driving alone: these activities remain the social norm in our societies [23]. Social norms can make any type of behaviour stable; they can be contrary to the collectively optimal outcome, and in fact reinforce suboptimal behaviour. Examples are norms that promote blood feuds [73], hate speech [74], compliance with protection rackets [75], corruption [76] or female genital mutilation [45].

How can we promote positive change when social norms are contrary to and, in fact, reinforce unsustainable behaviour? Public service announcements and educational efforts that rely on normative messages to alleviate problematic conduct can be undermined by the perception that the behaviour to be changed is common (i.e. by empirical expectations that suggest otherwise). An answer to this question will require studying norms that sustain socially undesirable behaviour (e.g. [77,78]), and particularly how to change them, e.g. through the framework of trending norm interventions [79]. An understanding of the emergence, stabilization and change of bad norms facilitates the development of targeted and effective interventions.

A second major limit to using social norms to address large-scale collective action problems is that under certain conditions they can lose their strength and become inefficient. The strength of social norms has been shown, for example, to be quickly eroded when new public information arrives [80]. If norm change indeed follows tipping points dynamics, erosion of social norms may result in fast (undesirable) changes in behaviour, when (slight) erosion brings expectations below the tipping point. Future research is required to understand how conditions that may lead to the erosion of social norms may interfere with the formation and stability of tipping points.

Finally, it is tempting to conclude that at least strong, stable social norms sustaining socially desirable behaviour can be the solution. Yet, norms that are too strong may introduce other externalities that are likewise inefficient. For example, cultures with very strong social norms often become repressive [16]. Moreover, when it comes to innovation, resourcefulness and creativity, loose cultures generally outperform cultures with stronger norms. Well-functioning societies require a carefully maintained equilibrium between these extremes [20]. Understanding how to meet and maintain these points of optimum efficiency at which social norms are efficient solutions to collective action problems is another important issue to be addressed in future studies of social norm change, for which empirically calibrated computational models are particularly suitable.

## 4. Conclusion

Social norms can play a key role in moving people to socially coordinate and take action against pressing societal issues such as pandemics, climate change and ecosystem destruction [1,2], but only if we understand how they motivate behaviour and how targeted interventions may strengthen or weaken them. While social norms have been of interest to scientists of a wide range of disciplines for many years, up until recently many studies considered the role of social norms only indirectly or implicitly, as post hoc explanations or by showing that the behavioural patterns are in line with the idea of social norms.

In this article, we have highlighted several important recent advances in the study of social norms that enable an unravelling of the effect social norms play in driving behaviour, to understand the processes of social norm change and ultimately to diagnose who or what type of expectations should be targeted through interventions to generate change. Most notably, these involve a clear and testable breakdown of collective social norms into micro-level social expectations [13], a theoretical framework to model and predict thresholds for social norm change [39,43] and methodological tools to test the causal effect of social norms [24,36].

Notwithstanding these important progresses, causal behavioural experiments and computational tipping point models have so far largely been developed independently of each other. We advocate a future research agenda that integrates these advances, for instance using empirically calibrated computational models to compare and contrast computational predictions to empirical data, unravel the mechanisms underlying the behavioural patterns, artificially test the effects of different interventions and scale up to understand processes of norm change in large societies. Empirically calibrated computational models are particularly suitable in the study of norm change, as change does not represent a linear process. Minorities can flip social expectations, but norms that are too strong may also prevent change. Such nonlinear, dynamic processes are difficult to measure using observational methods alone, whereas theoretical computational models lack clear applicability and generalizability.

By integrating empirical and computational models, social norm research may move beyond unequivocal praising of social norms as the missing link between rational self-interested behaviour and observed instances of cooperation [25] or as the explanation for (the lack of) social tipping [26]. It provides the toolkit to understand explicitly where, when and how social norms can be a solution to solve large-scale problems, but also to recognize their limits.

## Data Availability

This article has no additional data.

## References

[1] Bavel JJV *et al.* 2020 Using social and behavioural science to support COVID-19 pandemic response. Nat. Hum. Behav. **4**, 460-471. (10.1038/s41562-020-0884-z) 32355299

[2] Nyborg K *et al.* 2016 Social norms as solutions. Science **354**, 42-43. (10.1126/science.aaf8317) 27846488

[3] Nyborg K, Rege M. 2003 On social norms: the evolution of considerate smoking behavior. J. Econ. Behav. Organ. **52**, 323-340. (10.1016/S0167-2681(03)00031-3)

[4] Sparkman G, Walton GM. 2017 Dynamic norms promote sustainable behavior, even if it is counternormative. Psychol. Sci. **28**, 1663-1674. (10.1177/0956797617719950) 28961062

[5] Lapinski MK, Zhuang J, Koh H, Shi J. 2017 Descriptive norms and involvement in health and environmental behaviors. Communic. Res. **44**, 367-387. (10.1177/0093650215605153)

[6] Jachimowicz JM, Hauser OP, O'Brien JD, Sherman E, Galinsky AD. 2018 The critical role of second-order normative beliefs in predicting energy conservation. Nat. Hum. Behav. **2**, 757-764. (10.1038/s41562-018-0434-0) 31406290

[7] Higgs S, Liu J, Collins EIM, Thomas JM. 2019 Using social norms to encourage healthier eating. Nutr. Bull. **44**, 43-52. (10.1111/nbu.12371)

[8] Gelfand MJ *et al.* 2021 The relationship between cultural tightness–looseness and COVID-19 cases and deaths: a global analysis. Lancet Planet. Heal. **5**, e135-e144. (10.1016/S2542-5196(20)30301-6) PMC7946418 33524310

[9] Goldberg M. et al. 2020 Social norms motivate COVID-19 preventive behaviors,’ pp. 1–6. (10.31234/osf.io/9whp4)

[10] Walker B *et al.* 2009 Looming global-scale failures and missing institutions. Science **325**, 7-8. (10.1126/science.1175325) 19745137

[11] Weber EU. 2015 Climate change demands behavioral change: what are the challenges? Soc. Res. (New York) **82**, 561-580.

[12] Conte R, Andrighetto G, Campennì M. 2014 Minding norms: mechanisms and dynamics of social order in agent societies. Oxford, UK: Oxford University Press.

[13] Bicchieri C. 2006 The grammar of society: the nature and dynamics of social norms. Cambridge, UK: Cambridge University Press.

[14] Ostrom E. 2000 Collective action and the evolution of social norms. J. Econ. Perspect. **14**, 137-158. (10.1257/jep.14.3.137)

[15] Horne C, Mollborn S. 2020 Norms: an integrated framework. Annu. Rev. Sociol. **46**, 467-487. (10.1146/annurev-soc-121919-054658)

[16] Gelfand MJ *et al.* 2011 Differences between tight and loose cultures: a 33-nation study. Science **332**, 1100-1104. (10.1126/science.1197754) 21617077

[17] Biel A, Thøgersen J. 2007 Activation of social norms in social dilemmas: a review of the evidence and reflections on the implications for environmental behaviour. J. Econ. Psychol. **28**, 93-112. (10.1016/j.joep.2006.03.003)

[18] Cialdini RB, Reno RR, Kallgren CA. 1990 A focus theory of normative conduct: recycling the concept of norms to reduce littering in public places. J. Pers. Soc. Psychol. **58**, 1015-1026. (10.1037//0022-3514.58.6.1015)

[19] Elster J. 1989 The cement of society: a survey of social order. Cambridge, UK: Cambridge University Press.

[20] Gelfand MJ. 2018 Rule makers, rule breakers: how culture wires our minds, shapes our nations and drive our differences. Robinson.

[21] Feinberg J. 2014 Rights, justice, and the bounds of liberty. Princeton, NJ: Princeton University Press.

[22] Fehr E, Gächter S. 2000 Cooperation and punishment in public goods experiments. Am. Econ. Rev. **90**, 980-994. (10.1257/aer.90.4.980)

[23] Sparkman G, Howe L, Walton G. 2020 How social norms are often a barrier to addressing climate change but can be part of the solution. Behav. Public Policy **5**, 528-555. (10.1017/bpp.2020.42)

[24] Szekely A, Lipari F, Antonioni A, Paolucci M, Sánchez A, Tummolini L, Andrighetto G. 2021 Collective risks change social norms and promote cooperation: evidence from a long-term experiment. Nat. Commun. **12**, 5452. (10.1038/s41467-021-25734-w) 34526490 PMC8443614

[25] Bell DC, Cox ML. 2015 Social norms: do we love norms too much? J. Fam. Theory Rev. **7**, 28-46. (10.1111/jftr.12059) 25937833 PMC4412469

[26] Bicchieri C. 2017 Norms in the wild: how to diagnose, measure, and change social norms. New York, NY: Oxford University Press.

[27] Bianchi F, Squazzoni F. 2015 Agent-based models in sociology. Wiley Interdiscip. Rev. Comput. Stat. **7**, 284-306. (10.1002/wics.1356)

[28] Legros S, Cislaghi B. 2020 Mapping the social-norms literature: an overview of reviews. Perspect. Psychol. Sci. **15**, 62-80. (10.1177/1745691619866455) 31697614 PMC6970459

[29] Bicchieri C, Xiao E. 2009 Do the right thing but only if others do so. J. Behav. Decis. Mak. **22**, 191-208.

[30] Paluck EL. 2009 What's in a norm? Sources and processes of norm change. J. Pers. Soc. Psychol. **96**, 594-600. (10.1037/a0014688) 19254106

[31] Paluck EL, Shepherd HS. 2012 The salience of social referents: a field experiment on collective norms and harassment behavior in a school social network. J. Pers. Soc. Psychol. **103**, 899-915. (10.1037/a0030015) 22984831

[32] Henrich J *et al.* 2006 Costly punishment across human societies. Science **312**, 1767-1770. (10.1126/science.1127333) 16794075

[33] Fehr E, Fischbacher U. 2004 Third-party punishment and social norms. Evol. Hum. Behav. **25**, 63-87. (10.1016/S1090-5138(04)00005-4)

[34] Fehr E, Schurtenberger I. 2018 Normative foundations of human cooperation review-article. Nat. Hum. Behav. **2**, 458-468. (10.1038/s41562-018-0385-5) 31097815

[35] Schram A, Charness G. 2015 Inducing social norms in laboratory allocation choices. Manage. Sci. **61**, 1531-1546. (10.1287/mnsc.2014.2073)

[36] Krupka EL, Weber RA. 2013 Identifying social norms using coordination games: why does dictator game sharing vary? J. Eur. Econ. Assoc. **11**, 495-524. (10.1111/jeea.12006)

[37] Bicchieri C, Chavez A. 2010 Behaving as expected: public information and fairness norms. J. Behav. Decis. Mak. **23**, 161-178. (10.1002/bdm.648)

[38] Burks SV, Krupka EL. 2012 A multimethod approach to identifying norms and normative expectations within a corporate hierarchy: evidence from the financial services industry. **58**, 203-217.

[39] Centola D. 2021 Change: how to make big things happen. London, UK: John Murray (Publishers).

[40] Strimling P, De Barra M, Eriksson K. 2018 Asymmetries in punishment propensity may drive the civilizing process. Nat. Hum. Behav. **2**, 148-155. (10.1038/s41562-017-0278-z)

[41] Nardini G, Rank-Christman T, Bublitz MG, Cross SNN, Peracchio LA. 2021 Together we rise: how social movements succeed. J. Consum. Psychol. **31**, 112-145. (10.1002/jcpy.1201)

[42] Bicchieri C, Funcke A. 2018 Norm change: trendsetters and social structure. Soc. Res. (New York) **85**, 1-21.

[43] Granovetter MS. 1978 Threshold models of collective behavior. Am. J. Sociol. **83**, 1420-1443. (10.1086/226707)

[44] Andreoni J, Nikiforakis N, Siegenthaler S. 2021 Predicting social tipping and norm change in controlled experiments. Proc. Natl Acad. Sci. USA **118**, e2014893118. (10.1073/pnas.2014893118) 33859043 PMC8072257

[45] Efferson C, Vogt S, Elhadi A, Ahmed HEF, Fehr E. 2015 Female genital cutting is not a social coordination norm. Science **349**, 1446-1447. (10.1126/science.aaa7978) 26404811

[46] Xie J, Sreenivasan S, Korniss G, Zhang W, Lim C, Szymanski BK. 2011 Social consensus through the influence of committed minorities. Phys. Rev. E **84**, 011130. (10.1103/PhysRevE.84.011130) 21867136

[47] Baronchelli A, Felici M, Loreto V, Caglioti E, Steels L. 2006 Sharp transition towards shared vocabularies in multi-agent systems. J. Stat. Mech. Theory Exp. **6**, P06014. (10.1088/1742-5468/2006/06/P06014)

[48] Niu X, Doyle C, Korniss G, Szymanski BK. 2017 The impact of variable commitment in the Naming Game on consensus formation. Sci. Rep. **7**, 1-11. (10.1038/srep41750) 28150714 PMC5288711

[49] Iacopini I, Petri G, Baronchelli A, Barrat A. 2021 Vanishing size of critical mass for tipping points in social convention. See http://arxiv.org/abs/2103.10411.

[50] Centola D, Baronchelli A. 2015 The spontaneous emergence of conventions: an experimental study of cultural evolution. Proc. Natl Acad. Sci. USA **112**, 1989-1994. (10.1073/pnas.1418838112) 25646462 PMC4343158

[51] Centola D, Becker J, Brackbill D, Baronchelli A. 2018 Experimental evidence for tipping points in social convention. Science **360**, 1116-1119. (10.1126/science.360.6393.1082-d) 29880688

[52] Keuschnigg M, Lovsjö N, Hedström P. 2018 Analytical sociology and computational social science. J. Comput. Soc. Sci. **1**, 3-14. (10.1007/s42001-017-0006-5) 31930176 PMC6936355

[53] Dempsey RC, McAlaney J, Bewick BM. 2018 A critical appraisal of the social norms approach as an interventional strategy for health-related behavior and attitude change. Front. Psychol. **9**, 1-16. (10.3389/fpsyg.2018.02180) 30459694 PMC6232455

[54] Conte R, Paolucci M. 2014 On agent-based modeling and computational social science. Front. Psychol. **5**, 1-9. (10.3389/fpsyg.2014.00668) 25071642 PMC4094840

[55] Lazer DMJ *et al.* 2020 Computational social science: obstacles and opportunities. Science **369**, 1060-1062. (10.1126/science.aaz8170) 32855329

[56] Zhang H, Vorobeychik Y. 2019 Empirically grounded agent-based models of innovation diffusion: a critical review. Artif. Intell. Rev. **52**, 707-741. (10.1007/s10462-017-9577-z)

[57] Poteete AR, Janssen MA, Ostrom E. 2010 Working together: collective action, the commons, and multiple methods in practice. Princeton, NJ: Princeton University Press.

[58] Campbell DT. 1974 Downward causation’ in hierarchically organised biological systems. In Studies in the philosophy of biology, pp. 179-186. London, UK: Palgrave.

[59] Castelfranchi C. 1998 Simulating with cognitive agents: the importance of cognitive emergence. In MABS (eds JS Sichman, R Conte, N Gilbert), pp. 26-44. Berlin, Germany: Springer.

[60] Li X, Molleman L, van Dolder D. 2021 Do descriptive social norms drive peer punishment? Conditional punishment strategies and their impact on cooperation. Evol. Hum. Behav. **42**, 469-479. (10.1016/j.evolhumbehav.2021.04.002)

[61] Andrighetto G, Brandts J, Conte R, Sabater-Mir J, Solaz H, Villatoro D. 2013 Punish and voice: punishment enhances cooperation when combined with norm-signalling. PLoS ONE **8**, 1-8. (10.1371/journal.pone.0064941) PMC3680389 23776441

[62] Szekely A, Andrighetto G, Payette N, Tummolini L. 2020 Aggression, conflict, and the formation of intimidating group reputation. Soc. Psychol. Q. **83**, 70-87. (10.1177/0190272519882389)

[63] Bravo G, Squazzoni F, Boero R. 2012 Trust and partner selection in social networks: an experimentally grounded model. Soc. Netw. **34**, 481-492. (10.1016/j.socnet.2012.03.001)

[64] Taghikhah F, Filatova T, Voinov A. 2021 Where does theory have it right? A comparison of theory-driven and empirical agent based models. Jasss **24**, 4. (10.18564/jasss.4573)

[65] Realpe-Gómez J, Andrighetto G, Nardin LG, Montoya JA. 2018 Balancing selfishness and norm conformity can explain human behavior in large-scale prisoner's dilemma games and can poise human groups near criticality. Phys. Rev. E **97**, 1-22. (10.1103/PhysRevE.97.042321) 29758626

[66] Balliet D, van Lange PAM. 2013 Trust, punishment, and cooperation across 18 societies: a meta-analysis. Perspect. Psychol. Sci. **8**, 363-379. (10.1177/1745691613488533) 26173117

[67] Eriksson K. et al. ‘Responses to Rule Breakers across 57 Societies.’

[68] Harrington JR, Gelfand MJ. 2014 Tightness-looseness across the 50 united states. Proc. Natl Acad. Sci. USA **111**, 7990-7995. (10.1073/pnas.1317937111) 24843116 PMC4050535

[69] Jackson JC, Gelfand M, Ember CR. 2020 A global analysis of cultural tightness in non-industrial societies. Proc. R. Soc. B **287**, 20201036. (10.1098/rspb.2020.1036) PMC7423486 32605518

[70] Roos P, Gelfand M, Nau D, Lun J. 2015 Societal threat and cultural variation in the strength of social norms: an evolutionary basis. Organ. Behav. Hum. Decis. Process. **129**, 14-23. (10.1016/j.obhdp.2015.01.003)

[71] De S, Nau DS, Pan X, Gelfand MJ. 2018 ‘Tipping Points for Norm Change in Human Cultures’. In Social, cultural, and behavioral modeling. SBP-BRiMS 2018. Lecture notes in computer science, vol. 10899 (eds R Thomson, C Dancy, A Hyder, H Bisgin), pp. 61-69. Cham, Switzerland: Springer International Publishing.

[72] Tankard ME, Paluck EL. 2016 Norm perception as a vehicle for social change. Soc. Issues Policy Rev. **10**, 181-211. (10.1111/sipr.12022)

[73] Grutzpalk J. 2002 Blood feud and modernity. J. Class. Sociol. **2**, 115-134. (10.1177/1468795(02002002854)

[74] Álvarez-Benjumea A, Winter F. 2018 Normative change and culture of hate: an experiment in online environments. Eur. Sociol. Rev. **34**, 223-237. (10.1093/esr/jcy005)

[75] Andrighetto G, Grieco D. 2020 Peer effects on compliance with extortive requests. PLoS ONE **15**, 1-17. (10.1371/journal.pone.0231879) PMC7182209 32330154

[76] Bicchieri C, Rovelli C. 1995 Evolution and revolution: the dynamics of corruption. Ration. Soc. **7**, 201-224. (10.1177/1043463195007002007)

[77] Abbink K, Gangadharan L, Handfield T, Thrasher J. 2017 Peer punishment promotes enforcement of bad social norms. Nat. Commun. **8**, 1-8. (10.1038/s41467-017-00731-0) 28931829 PMC5607004

[78] Centola D, Willer R, Macy M, Centola D, Willer R, Macy M. 2005 The emperor's dilemma: a computational model of self-enforcing norms. Am. J. Sociol. **110**, 1009-1040. (10.1086/427321)

[79] Mortensen CR, Neel R, Cialdini RB, Jaeger CM, Jacobson RP, Ringel MM. 2019 Trending norms: a lever for encouraging behaviors performed by the minority. Soc. Psychol. Personal. Sci. **10**, 201-210. (10.1177/1948550617734615)

[80] Bursztyn L, Egorov G, Fiorin S. 2020 From extreme to mainstream: the erosion of social norms. Am. Econ. Rev. **110**, 3522-3548. (10.1257/AER.20171175)

